# Navigating the ‘moral hazard’ argument in synthetic biology’s application

**DOI:** 10.1093/synbio/ysae008

**Published:** 2024-05-23

**Authors:** Christopher Hunter Lean

**Affiliations:** ARC Centre of Excellence in Synthetic Biology, Macquarie University, Sydney, NSW, Australia; Department of Philosophy, Faculty of Arts, Macquarie University, Sydney, NSW, Australia

**Keywords:** environmental remediation, moral hazard, carbon sequestration, technology ethics, Playing God

## Abstract

Synthetic biology has immense potential to ameliorate widespread environmental damage. The promise of such technology could, however, be argued to potentially risk the public, industry or governments not curtailing their environmentally damaging behavior or even worse exploit the possibility of this technology to do further damage. In such cases, there is the risk of a worse outcome than if the technology was not deployed. This risk is often couched as an objection to new technologies, that the technology produces a moral hazard. This paper describes how to navigate a moral hazard argument and mitigate the possibility of a moral hazard. Navigating moral hazard arguments and mitigating the possibility of a moral hazard will improve the public and environmental impact of synthetic biology.

## 1. Introduction

One of the most promising applications of synthetic biology is its use to mitigate environmental damage (1). Current projects include the engineering of capacities for the removal of pollutants from the environment (e.g. plastic degradation (2, 3)), the use of synthetic biology to engineer wild populations for their protection (e.g. engineered Chestnut-blight resistance in Chestnuts (4)) and climate change mitigation (e.g. engineering biofuels and carbon sequestration (5, 6)). There is a common goal in all these projects to use cutting-edge biotechnology to remediate environmental destruction driven by human actions. However, technologically rectifying this environmental damage does not address its initial cause: human action. If such damage-mitigating technology leads institutions or the public to believe that they do not need to curtail their environmentally destructive behavior (EDB), or they can increase this behavior in response to this mitigation, then it could yield worse environmental outcomes.

This objection is known as the ‘moral hazard argument’. To be specific: ‘a Moral hazard occurs when the actions of one party absorb the potential negative consequences of the behavior of another party/parties, resulting in an inefficient increase in risk-taking behavior’ (7) (Ben (8) identifies sixteen different ‘moral hazard’ objections to geoengineering for climate change. To narrow the scope of this paper, I am using this precisification of the objection, primarily focusing on situations that lead to net environmental damage compared to the situation where the technology was not released). If the damage mitigated by a technology is outweighed by increasingly risky actions of those who cause environmental damage, then the introduction of that technology will yield worse outcomes. The exploitation of the presence of the technology may be conscious but could also be due to an overestimation of its effectiveness or the assumption that new technologies will emerge to mitigate any further environmental damage. I include cases in which a technology excuses a polluter from effectively mitigating polluting practices, where it is probable their action would have otherwise changed.

This paper provides a guide to the moral hazard argument and advises how to engage in environmental mitigation effectively and ethically. Moral hazard arguments have recently been influential in critiquing new technologies for addressing climate change (7, 8). Negative emissions technologies, including those developed from synthetic biology, have been argued to create moral hazards as the act of removing carbon from the atmosphere through new technology could foster further climate change, as it absorbs the negative consequences of climate change without addressing its root cause, and ultimately could disincentivize emissions reduction (9).

This argument equally applies to the development of synthetic biology for other forms of environmental remediation. For example, many have argued against the use of biotechnology to engineer ecological proxies for extinct species because the production of such populations could undermine current conservation measures by making extinction appear impermanent (10). The removal of pollutants through synthetic biology could be argued to risk a moral hazard, as it could disincentivize investment in reducing the production and release of pollutants into the environment by manufacturers and mining companies by reinforcing the expectation that environmental damage can be addressed after the act. Given the general applicability of this argumentative structure, synthetic biology practitioners should know how to navigate this argument and identify whether it applies to their research.

Moral hazards are features of the socioecological system that influence the environment. There is a ‘risk-response feedback loop’ between those providing environmental policies (inclusive of new technologies) and individuals and social institutions responding to those policies (11). The package of policies affects biophysical systems through direct environmental effects and indirect sociological effects (11, p. 3). The interactions between environmental and social factors can be dynamic and complex. This feedback loop ultimately determines the net impact of new technology on the environment. Understanding novel technologies as being part of a wider system, for which there can be negative feedback relations is critical for considering how to avoid moral hazard. While it is not possible to predict the dynamics of the range of socioecological systems that new synthetic biology-based technology will be introduced we can identify some key factors that can cause a moral hazard dynamic.

Two key factors govern whether a moral hazard argument is relevant. The first is a behavioral assumption that those observing the employment of synthetic biology in environmental mitigation will adjust their behavior to exploit the absorption of risk. The second is that the potential exploitation will result in net environmental damage. Finally, one should be wary of objections purporting to be moral hazard arguments that are intrinsic objections to the research. In such cases, the net environmental impact is irrelevant to responding to the argument, and different forms of engagement are required.

## 2. The behavioral assumption

For a new technology to be morally hazardous, the second party (those responding to the new technology) must either curtail their projected reduction of EDB or increase their EDB by exploiting the risk compensation created by the novel technology. If the novel technology is implemented and the EDB continues to be curtailed, then there is no exploitation of the risk compensation, and it will consequently yield positive environmental outcomes. For a synthetic biology-based technology to effectively mitigate environmental destruction, then, there should be enquiry before the release of the technology into whether secondary parties will exploit the risk compensation of this new technology. For example, if we create insects that produce fungal laccases that can remove bisphenols, industrial dyes, phenols, etc. (12), we should ask whether companies would then reduce their investment in reducing the emission of these pollutants. Further, we must ask how we can ensure such behavior is unlikely to occur.

### 2.1 Can we predict moral hazard behavior?

It is difficult to know the behavioral response to a novel technology before it is released. To gain insight, we can either consider case studies of previously implemented environmental mitigation technologies to identify whether they were exploited by second-party actors. Alternatively, we could survey second-party actors on their predicted actions. These can be surveys of their first-person predicted actions or designed to extract revealed preferences.

Moral hazards have been widely researched in other fields, particularly insurance where there has been a long history of enquiring as to whether insurance leads to more risky behavior (13). The empirical literature indicates that moral hazard exists in some insurance contexts but not others (14). For example, automobile insurance is correlated with traffic fatalities (‘The magnitude of this moral hazard effect is potentially large: a 2 percent increase in the number of fatalities for each percentage point decrease in the number of uninsured motorists’ (15, p. 388)), particularly no-fault liability insurance (15). Moral hazard appears to, therefore, increase when significant external costs for increasing risk exposure are removed, in this case, the removal of the chance of being sued for being at fault in an accident. In contrast, there is no evidence of a connection between seat belt legislation and riskier driving (16). In the case of medical insurance, one of the most empirically researched cases, there is insufficient empirical evidence to conclude it is a significant factor (17, p. 369). If there is a generalizable conclusion to be gained from this research about the ubiquity of moral hazards, it is that moral hazards cannot be assumed and are specific to local contexts.

Given the inconclusive nature of the evidence for the frequency of moral hazards in other fields, dedicated studies of the historical impact of environmental technologies are required to discern the extent to which environmental technologies yield unscrupulous behavior by second parties. There is difficulty in identifying good case studies as we are forced to compare the trajectory of environmental actions in the actual case of technological implementation against the possible trajectory of environmental action in the hypothetical case, where the technology was not known to exist. Significant cases of moral hazard in environmental technology, however, appear to exist. One example is Carbon Capture and Storage (CCS), particularly its development under the name ‘Clean Coal’. Governments, including the UK, USA, Germany and Australia, have used the concept of Clean Coal to justify the continued use of inefficient coal-fired power plants or the establishment of new ones, rather than transitioning to lower-emission energy production (18). For example, CCS technology was tacitly used to justify the continued use of the Hazelwood Power Station, the most inefficient plant in the Organisation for Economic Co-operation and Development, while the CCS technology was only able to sequester 0.05% of the plant’s emissions (19).

The potential of a technology and its impact, particularly in its initial deployment, can be distinct. Despite the negative environmental consequences associated with CCS technology in coal plants, there is potential for other forms of CCS to mitigate emissions. Specifically, other forms of CCS are being developed to support alternative energy production methods, such as bioenergy and carbon capture storage power plants. Synthetic biology is a major contributor to the creation of these new plants through the engineering of efficient biofuels. These developments have the potential to significantly reduce climate change (20). However, critics argue that these plants will not be sufficiently efficient to markedly reduce emissions (9). It is important to consider the historical deployment and reception of related environmental technologies to navigate public perceptions and avoid exploitation by vested interests.

Interpreting implications from case studies is contentious, and it is critical to proactively gain information about the likely reception of a novel technology. Surveying secondary parties and stakeholders about their likely actions upon its release is the primary method used to predict responses to a novel technology. One of the most scrutinized cases of suspected moral hazard in the use of novel technology is geoengineering for climate change mitigation (21). Interestingly, surveys of the public on geoengineering indicate that the prospect of using this technology either does not affect or increases their commitment to emission reductions (7, 22, 23). Pairing these results with those from studies of insurance ‘indicates’ that moral hazards are not as ubiquitous as some would imagine. Further, overly anticipating moral hazards, due to a lack of preliminary research, can result in worse environmental outcomes as it can suppress the development and use of effective technologies (24). Given this, scientists should defend research directions that some argue should be rejected due to a chance of moral hazard when there is little evidence for moral hazard being offered. Preliminary research on the influence of new technology on behavior is valuable as both underestimation and overestimation of the risk of moral hazard can lead to a negative impact.

Surveys may not represent all the factors that shape actors’ responses to the new technology and are fallible. Testimony from surveys about uncertain events is subject to many well-known biases, which may undermine the validity of survey data. Further, we need to trust their testimony. There may be incentives to misrepresent their future actions if they intend to exploit the environmental risk mitigation resulting from the new technology. Many environmental decisions are made by institutions, not just individuals and there may be institutional incentives that could produce outcomes that differ from what is indicated in surveys, even if those surveys are given to those within that institution (11). Neither case studies nor surveys can provide an unequivocal indication of when and whether a moral hazard will arise. Despite this, their cautious use will act as an important indicator of whether moral hazard will arise and will help direct effective stakeholder engagement.

### 2.2 Pre-empting moral hazard behavior

The evidence for the existence and frequency of moral hazards is difficult to parse. Given this, the evidence for effective actions is even more difficult to discern. The insurance markets show that moral hazards are not ubiquitous, but a critical feature is that insurance companies actively design policies to minimize the chance of moral hazards. I will translate some common methods utilized by insurance companies to indicate some initial principles for minimizing moral hazards, I will draw from Ben-Shahar and Logue’s (25) discussion of these methods. We can also look to previous discussions of how to minimize moral hazards in particular technologies, such as geoengineering (7, 26, 27), or general rules for how to avoid exploitation or strategically communicate to stakeholders. These will provide some methods for how to avoid moral hazards that will be summarized in Box 1.

Box 1. Pre-empting moral hazard.

**Engagement**

– Establish upfront commitments to environmental damage mitigation through public statements or binding agreements.
– Avoid working for or with companies and funding bodies that show no indication of wanting to reduce the emission of pollutants.
– Establish monitoring procedures for identifying the impact of the new technology including its possible exploitation before the release of the technology and communicate this.

**Communication**

– Communicate to the public that novel technology cannot outcompete increasing environmental damage.
– Communicate to governments how to most effectively legislate novel technology to ensure its effectiveness and avoid exploitation.
– Communicate to businesses the risks of relying solely on novel technology compared to environmental damage mitigation.

**Research**

– Research how the novel technology integrates with existing methods of environmental mitigation to identify best practices.

Insurance companies use a range of methods to minimize the change of moral hazards occurring (25). Differentiated premiums are used where careful existing insurance holders are given more favorable insurance premiums, so both parties must engage in upfront costs for entering the contract dependent on their risk exposure. In the environmental case, variation in technology investment and deployment could be contingent on the investment and communication of the direct means of reducing EDB (27). This needs to be done by both the scientists and institutions that fund this research. Upfront commitments to environmental damage mitigation could take the form of public statements or even binding agreements with relevant parties that they are committed to the continued reductions in producing pollutants. This is particularly important if the research is funded by polluting parties.

A major method for insurance companies is to refuse to insure individuals’ records of high-risk behavior. In working on environmental technologies this could translate to working with funding bodies, where there is an established record of effectively working on environmental damage mitigation. Providing research support for those with little record of committing to environmental damage mitigation can increase the risk of exploitation.

External oversight is critical for the punishment of those who would exploit a new technology. Insurance companies will provide oversight of policyholders through audits or even monitoring clients’ behavior and influencing the legal frameworks that apply to citizens. While scientists cannot enforce laws, they can foster the punishment of those who ignore laws, public statements or binding agreements. This is best done through establishing clear monitoring protocols so the environmental impact of the technology and whether other actors are exploiting it can be communicated to the government and the public. This is important, regardless, given that monitoring is crucial for ensuring the technology has the intended impact.

Insurance coverage is often based on deductibles and copayments, meaning those insured will still receive a cost for exposing themselves to risk. This is a form of loss sharing, so the insurance company does not shield the policyholder from all risk exposure. In the case of environmental damage mitigation, given the source of environmental damage is ongoing the risk exposure remains (26). Any new technology is only a partial solution to the issue, of which traditional mitigation of EDB must be part. Communicate that novel technology cannot outcompete increasing environmental damage. The fact that risks are continuous and shared, as long as pollutants/environmental loss occurs, even in the case of new technologies ameliorating risks must be forcefully communicated.

Coaching safer conduct of those insured is a major method by which moral hazards are reduced. This has a clear analogy for those developing technologies where their technology must be communicated in the context of the existing methods for environmental damage mitigation and emphasizing the importance of using these measures in conjunction with the new technology. Novel technologies exist within a package of socio-political policies for addressing environmental damage that must include mitigation by significant actors and institutional incentives for ongoing environmental damage mitigation. Mitigation is better than remediation.

Further insurance companies influence government regulation and safety methods. Equally, those in environmental technologies can invest efforts into not just the technology itself but also how this technology integrates with existing methods for mitigating EDB and legislation that ensures that environmental damage is reduced. New techniques for environmental remediation need to be integrated with existing methods of preventing environmental damage to create new best practices that should be widely communicated. This includes speaking to governments on legislating novel technology to ensure its effective use.

This is not of course a comprehensive list but should provide some guidance on how to avoid moral hazards before the implementation of the technology. These solutions may conflict with the incentives of selling the potential of new technology and identifying funding, but if the aim is to create technologies that produce positive environmental outcomes rather than technologies that sell, then the risk of engagement with stakeholders is merited.

## 3. The net impact

Moral hazard objections to new technology often overemphasize the significance of increased EDB, due to the moral objectionability of this behavior. But if the goal of this technology is to reduce the negative impact of human activity on the environment, then behavior cannot be considered in isolation. Even if the release of a technology precipitates an increase in risk-taking behavior by second parties, a novel technology could produce net positive environmental outcomes if it is highly effective. Therefore, accurate predictions are needed not just for the likelihood of the exploitation of this technology but also for the effectiveness of the exploitation and the effectiveness and risks of employing this technology.

Even in cases of moral hazard behavior, we must consider whether those exploiting the presence of the technology have the power to create a significant impact. In the previous section, I distinguished that moral hazard behavior may be institutional or individual. There is a question of not just who is likely to make an impact but the causal efficacy of that impact on the environment. When we consider the case of CCS technology, the exploitation of this technology was done by governments and large businesses, not the public. When we consider climate change more broadly, 100 companies are responsible for 71% of global emissions since 1988 (28). Given businesses and governments are the ones with the capital to deploy and/or exploit new environmental technologies and the largest contributors to EDB they are the ones with the largest capacity to exploit the presence of new technologies. The degree to which businesses and governments can efficiently and effectively influence the environment means that it can be wise to prioritize pre-emptive engagement with them. The public is still important, particularly given they can influence businesses and government, forcing ethical action. Further responsible research and innovation must be in light of and in dialogue with public opinion (29, 30). But consideration must always be made for which course of action will yield the best results for the environment so care should be directed to those with the most capacity to cause environmental destruction.

Determining the positive effect of emerging technologies as compared to existing practices involves weighing predictions, and projected risks, against reality. While synthetic biology promises uniquely effective means for processing environmental contaminants *in situ*, there are low probability, but significant risks, found in engineering novel autonomous life forms to perform a role that can be done by established means. Existing techniques for preventing environmental damage will be preferable to new technologies when they are equitably effective in rectifying environmental damage if the new technology holds further unknown or low-probability, high-impact risks. This is a version of the precautionary principle (31). A well-developed literature exists on the merit of the precautionary principle in synthetic biology, and the overzealous application of it could forestall significant technologies that could have immense positive effects (32). An example of this is the research into mRNA vaccines, which could have been prevented through an overzealous application of the precautionary principle in the 1990s but has been central to the global COVID-19 response. (The rapid deployment and acceptance of mRNA vaccines were due to factors including having well-established vaccine platforms and experience with prior human coronaviruses but mainly public will providing funding, volunteers for vaccine testing and openness to using new vaccines (33). My own belief is that this was due to the cost’s individuals suffered due to lockdowns and disease making them more open to risk. We want to avoid such levels of widespread individual suffering being necessary to bring about the acceptance of environmental remediation technologies.) Precaution is warranted in the form of protocols and biosecurity frameworks to account for the range of risks created by synthetic biology (34). The objection that novel technology brings about novel risks compared to traditional mitigation techniques does indicate we should prefer traditional methods, but only when we hold fixed the efficacy of both in addressing environmental damage. When new technology becomes more efficient and implementable, these low-probability, high-impact risks become outweighed by the risks of inaction to environmental damage.

An important consideration for net impact is lag times in the rollout and optimization of new technologies. Any new technology takes time to optimize as early versions typically will not yield high efficiencies. This makes timing the technology’s release and communicating its effectiveness risky with respect to moral hazard. If a novel technology fosters an increase in EDB, then the early release of a technology before it has been optimized can lead to a significant negative net environmental impact. This can be true even if the optimized version of that technology could create a positive environmental effect due to the time needed to offset the originally inflicted environmental damage. Equally, selling technology as being more effective than it currently is could lead to higher EDB on the unjustified assumption this technology can fix the inflicted damage. So, to ensure that environmental mitigation is effective one should (i) honestly communicate the effectiveness of the technology to all parties who can influence environmental damage and (ii) delay the release of inefficient versions of environmental damage-mitigating technologies when there is reason to believe some may exploit the presence of this technology.

## 4. Intrinsic objections

Some may oppose a novel technology or policy as they explicitly think there is a moral component to the act that is being mitigated. They will then object to the technology solely due to their moral objection rather than due to a fear of negative outcomes. For example, some have argued that using geoengineering to avoid climate change is morally wrong as it is an act of control and hubris toward nature (i.e. the intervention is immoral) or it allows humanity to continue its consumptive behavior (i.e. the intervention fosters immoral behavior) (8). Scientists should be cautious navigating such arguments, as while these are a form of moral hazard, they require different responses to ensure the technology is accepted by stakeholders or will require reconsidering the use of such technology.

**Figure 1.**
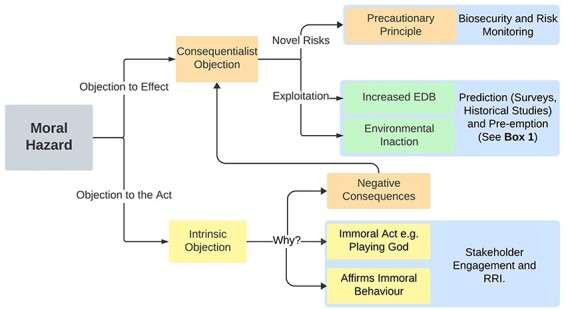
Decision tree for navigating moral hazard objections. The first stage of navigating moral hazard is identifying the content of the objection. Then we can identify routes for addressing these concerns through mitigating risk, justifying methods or adjusting/abandoning research goals and methods to align with stakeholder concerns (intrinsic objections = yellow; general consequentialist objections = orange; environmental consequentialist objections = green; responses to moral hazard objections = blue).

Opposition to the technology, irrespective of its demonstrated effectiveness, may appear in the guise of a moral hazard argument. This phenomenon could be quite common in the case of synthetic biology, where some in the public may oppose the technology because it is ‘Playing God’, which appears to constitute an objection that the intervention is immoral. Interestingly, when the public is pressed to explain ‘Playing God’ objections to synthetic biology they often cite fears of distinct quantifiable risks (35). There is an open question about what has precedence in the minds of the public, whether this is the *post hoc* rationalization of rejecting the act or the view that these acts have risks, and ‘Playing God’ is merely a description expressing these risks (which may vary across the public). If the objection is based on the fear of direct risks, then evidence of the safety and effectiveness of the technology may assuage these objections. If they reject the act as morally objectionable, evidence of the effectiveness of the intervention will not cause the public to accept the technology (Figure 1).

Similarly, objections may also be against any act of mitigation that could allow for an increase in EDB, regardless of the effectiveness of the technology or the actuality of the increased behavior. In these cases, the objection is that the intervention fosters or affirms immoral behavior. For example, engineering the decomposition of Trinitrotoluene (TNT) may lead to intrinsic objections as any mitigation of TNT use may be seen as tacitly permitting conflict (1).

In both cases of objecting to the act or arguing the technology fosters immoral behavior, significant research into the range of public opinions is critical for ethical research. Stakeholder engagement to normalize the technology and emotionally justify the technology may be effective in convincing the public of its value (36). Ultimately, however, scientists should be responsive to strong moral objections and in some instances, this would require abandoning certain projects as most responsible research and innovation frameworks would recommend moratoriums or abandoning this line of enquiry (29, 30).

Intrinsic objections stand outside the weight of good and ill effects, and social, political and moral engagement with stakeholders will be required to determine whether a technology can be ethically employed. The first step will be teasing apart whether moral objections are to the act or the outcome of the new technology as these yield different courses of action for deciding how to ethically implement new technology.

## 5. Conclusion

While the existence of a moral hazard is not a foregone conclusion for new technologies, it is wise to mitigate their possibility. Direct research into the likelihood of the exploitation of the developments emerging out of synthetic biology should be employed as part of ethical design. The effective navigation of moral hazard-based risk will require premeditated engagement with stakeholders to avoid the exploitation of environmental mitigation and honest communication of the effectiveness of the technology. These actions are in themselves preferable for open and civically engaged science and reinforce norms that regardless of the risk of moral hazards should be employed by those developing new technologies.

## Data Availability

Data sharing is not applicable to this article as no new data were created or analysed in this study.
